# Catheter-Directed Arterial Thrombolysis with a Low-Dose Recombinant Tissue Plasminogen Activator Regimen for Acute Lower Limb Ischemia—Results of the First Regional Registry of Acute Limb Ischemia in Romania

**DOI:** 10.3390/life14111516

**Published:** 2024-11-20

**Authors:** Sorin Barac, Roxana Ramona Onofrei, Octavian Barbu, Stelian Pantea, Cristina Pleșoianu, Ciprian Gîndac, Bogdan Timar, Andreea Luciana Rață

**Affiliations:** 1 Department of Vascular Surgery, “Victor Babes” University of Medicine and Pharmacy, 300041 Timișoara, Romania; sorin.barac@umft.ro (S.B.); octavian.barbu@umft.ro (O.B.); andreea.rata@umft.ro (A.L.R.); 2 “Pius Brînzeu” Clincal County Emergency Hospital, 300723 Timișoara, Romania; onofrei.roxana@umft.ro (R.R.O.); ciprian.gindac@umft.ro (C.G.); 3 Romanian Society for Vascular Pathology, 300633 Timișoara, Romania; 4 Department of Rehabilitation, Physical Medicine and Rheumatology, Research Centre for Assessment of Human Motion, Functionality and Disability, “Victor Babes” University of Medicine and Pharmacy, 300041 Timișoara, Romania; 5 Surgical Emergencies Department, “Victor Babes” University of Medicine and Pharmacy, 300041 Timișoara, Romania; 6 The Academy of Economic Studies, 010552 Bucharest, Romania; cristina.plesoianu@gmail.com; 7 Romanian Academy of Medical Sciences, 030167 Bucharest, Romania; 8 Anesthesiology and Intensive Care Department, “Victor Babes” University of Medicine and Pharmacy, 300041 Timișoara, Romania; 9 Second Department of Internal Medicine, “Victor Babes” University of Medicine and Pharmacy, 300041 Timișoara, Romania; bogdan.timar@umft.ro

**Keywords:** acute limb ischemia, catheter-directed arterial thrombolysis, limb salvage

## Abstract

Acute limb ischemia is a limb-threatening condition that is associated with a high degree of mortality and morbidity, with the latter related to acute kidney injury and rhabdomyolysis that can rapidly lead to multiple organ failure. The aim of this study was to assess the efficacy and safety of catheter-directed arterial thrombolysis in acute lower limb ischemia in the Department of Vascular Surgery, Timișoara, Romania. A total of 158 patients (114 males—72.15% and 44 females—27.85%) with symptoms of acute lower limb ischemia were admitted and treated with catheter-directed arterial thrombolysis following our protocol. The amputation-free survival rate at 1 month after the thrombolysis was 82.3%, and at 6 months it was 77.85%. The performance of additional procedures to obtain distal perfusion was predictive of an improved outcome at 30 days. The estimated survival rate at 6 months was 84.81% (SE 0.02). The mean survival time was 158.74 days. We recommend the usage of a thrombolytic regimen in patients with a life expectancy of more than 6 months, even in Rutherford stage IIb patients, if there is no major impairment in the sensorial and mobility function of the ischemic leg.

## 1. Introduction

Acute limb ischemia (ALI) is an important condition that can lead to limb loss if untreated. The systemic reflection of acute limb ischemia can lead to a high degree of morbidity and mortality (acute kidney disease, rhabdomyolysis, etc.).

It is well known that acute limb ischemia is caused either by embolism or by thrombosis (in situ thrombosis or bypass thrombosis), and in many cases it can be an association of both mechanisms.

Since 1962, balloon thrombo-embolectomy was the standard treatment for ALI caused by embolization [1], but nowadays, according to the 2020 ALI Guidelines, intra-arterial catheter-directed thrombolysis (CDT) can be performed in ALI with equivalent results to surgery [2,3,4,5]. Initially, thrombolysis was indicated in patients with Rutherford stages I or IIA [6], but there are several studies that showed that it can also be used in patients with Rutherford stage IIb [7,8,9,10,11]. Initially, thrombolytic therapy was used for patients with acute ischemia whose limb was not immediately threatened or who did not have a very rapid progression of symptoms. Ebben et al.’s systematic review demonstrated that thrombolysis can also be used in patients with Rutherford stage IIb and that the results are also good in patients with a motor deficit. Furthermore, when considering more severe ischemia, these patients may require higher doses of thrombolytic treatment [7]. Although there are several studies about intra-arterial CDT, the treatment protocol and the optimum dosage of the thrombolytic agent is still open to debate.

The aim of this retrospective study was to assess the efficacy and safety of catheter-directed arterial thrombolysis in acute lower limb ischemia as a new technique in the Romanian health system.

## 2. Materials and Methods

From May 2016 to February 2020, 570 patients with acute lower limb ischemia were admitted to the Department of Vascular Surgery of “Pius Brînzeu” County Clinical Emergency Hospital, Timișoara, Romania.

Fifty-two of them suffered major limb amputation per primam (9.12%) because of irreversible damage in the lower limb (no motility or sensibility), 39 patients (6.84%) were treated conservatively, 299 patients (52.45%) were treated with Fogarty catheter embolectomy, and the remaining 158 patients (27.71%) were treated with catheter-directed arterial thrombolysis.

A total of 158 patients (114 males—72.15% and 44 females—27.85%) with symptoms of acute lower limb ischemia were admitted and treated with catheter-directed arterial thrombolysis for acute lower limb ischemia. Acute limb ischemia was documented through the history, clinical examination, Doppler ultrasound, and CT Angio. CT Angio was performed in all patients as part of the diagnosis and in accordance with the ESVS Guidelines on Acute Limb Ischemia [5]. All patients were assigned to a Rutherford stage after admission.

The patients were included in the study based on their diagnosis and were excluded based on contraindications for the thrombolytic regimen, as stated in the ESVS Guidelines [5].

The treatment protocol was as follows:

- All patients had coagulation testing prior to thrombolytic administration (international normalized ratio—INR, activated partial thromboplastin test—aPTT, platelet count) and a set of paraclinical tests in order to have a complete assessment of the patient (hemogram, serum urea, serum creatinine, hepatic enzymes, creatin-kinase (CK), and creatin-kinase-MB (CK-MB);
- All patients had an ECG and a cardiologic assessment prior to the thrombolytic treatment;
- Heparin IV treatment was started for all patients prior to the thrombolytic therapy (with a bolus of 5000 IU and with an aPTT determination every 4 h and with targeted values of 60–70 s);
- All patients had urinary catheter placement for monitoring diuresis and were carefully monitored in terms of vital functions (arterial tension, O_2_ saturation, and cardiac rhythm);
- All patients received their usual treatment for associated pathologies;
- Access for the catheter was either femoral or brachial using the Merit Fountain Thrombolysis Catheter and Infusion System^®^ (Merit Medical, South Jordan, UT, USA);
- The dilution for the thrombolytic agent was 1:1;
- After the catheter placement, 10 mL of rt-PA (recombinant tissue plasminogen activator; Boehringer Ingelheim International GmbH, Ingelheim am Rhein, Germany) was infused as a bolus;
- 40 mL of continuous perfusion was administered to the intra-arterial catheter at a rate of 1 mL/h for no more than 24 h;
- Simultaneously, heparin was administered to the femoral or brachial sheath on continuous perfusion of 250 UI/mL/h;
- Control angiography and additional endovascular procedures were performed if necessary.

We administered this protocol in patients with different symptom onsets but for no longer than 21 days and with stable ischemia. All patients signed an informed consent form for the treatment and for the use of their clinical files under proper anonymization. The data were collected from the patients retrospectively under the GDPR laws. The study had the agreement of the Hospital Ethics Committee (no. 189/04.05.2020) under the EU GCP Directives, the International Conference of Harmonization of Technical Requirements for Registration of Pharmaceuticals for Human Use (ICH), and the Declaration of Helsinki.

We compared data from the patients treated with catheter-directed arterial thrombolysis with those treated through open surgery in terms of complications, major cardiac events, and major limb events. Follow-up of the patients was made at 30 days, 3 months, and 6 months. The follow-up parameters were the ankle brachial index (ABI) on the index leg (determined within the clinical guidelines’ indications) [12] and the appearance of complications because of the thrombolysis. The primary outcome was the amputation-free survival rate at 1 and 6 months. The secondary outcome was overall mortality at 6 months and an increase in the ABI.

### Statistical Analysis

The statistical analysis was performed with MedCalc version 19.7 (MedCalc Software Ltd., Ostend, Belgium). The data are expressed as means ± standard deviations (SDs) for the continuous variables with normal distribution and as medians [interquartile ranges] for the continuous variables without normal distribution. The categorical variables are expressed as counts and percentages. The overall survival rate and amputation-free survival rate were estimated using the Kaplan–Meier method. The differences between the groups were evaluated with a log-rank test. In order to identify the predictors of negative outcomes (major amputation and death), the variables (*p* < 0.2 in the univariate analysis) were entered into a Cox proportional hazard regression (forward model). An increase in the ankle brachial index was analyzed with the Friedman test. A *p*-value < 0.05 was considered statistically significant.

## 3. Results

One hundred and fifty-eight patients aged 24–87 years were included in the study (Table S1 in the Supplementary Materials). The baseline characteristics are detailed in Table 1. Similarly, Table 1 also shows the demographics of the patients treated with open surgery without embolectomy. It is worth mentioning that statistically significant differences were noted only in the presence of heart failure and in the presence of atrial fibrillation as an embolic risk factor in these patients. Both endovascular treatment and open surgery are optimal treatment options for acute limb ischemia with similar success rates. Thus, after the CT Angio analysis, the cases with ALI of clearly embolic origin were treated with Fogarty embolectomy, and the other ones were treated with endovascular procedures.

Symptom duration before admission for the thrombolysis group was less than 24 h in 30 patients (18.98%), 2–7 days in 60 patients (37.97%), 7–14 days in 30 patients (18.98%), and more than 14 days in 38 patients (24.05%). In the open surgery group, the data were as follows: less than 24 h in 108 patients (36.12%), 2–7 days in 143 patients (47.82%), 7–14 days in 31 patients (10.36%), and more than 14 days in 17 patients (5.68%). The differences between the groups were statistically significant for the periods of 2 to 7 days and more than 14 days. In the thrombolysis group, there were 18 cases (11.39%) of Rutherford stage I ischemia, 77 cases (48.74%) of Rutherford stage IIA, and 63 cases (39.87%) of Rutherford stage IIB. In the open surgery group, we had eight patients (2.67%) in stage I, 176 patients (58.86%) in stage IIA, and 115 patients (38.46%) in stage IIB.

The 18 patients in the thrombolysis group and the eight patients in the open surgery group with class I ischemia included in the study had distressing symptoms, and these were active people for whom the symptoms excessively affected their daily quality of life. Thus, after a thorough analysis of their symptoms and arterial tree, it was decided to treat them. The pre-interventional ankle brachial index was between 0 and 0.5 for both groups without statistically significant differences between the two groups. The mean time of thrombolysis was 25.82 ± 6.84 h, and the mean administered dose was 35.6 ± 6.88 mL. The approach was femoral in 87 cases (55.06%), of which 19 were through crossover (12.02%) and 71 were brachial (44.94%). In 66 cases (41.77%), additional procedures were performed to obtain sufficient distal perfusion (44 balloon dilatation angioplasties—balloon PTA)—66.67%; 10 angioplasties with stent placement (stent PTA)—15.15%; 10 bypasses—15.15%; and two hybrid interventions—3.03%).

In the open surgery group, we had three types of approaches: only femoral—280 patients (93.64%), only popliteal—8 patients (2.67%), and both femoral and popliteal—11 patients (3.67%).

In the thrombolysis group, hemorrhagic complications occurred in 33 cases (20.88%)—28 cases of hematoma (84.85%), two cases of digestive hemorrhage (6.06%), and three cases of cerebral hemorrhage (9.09%). There were 11 major bleeding complications (6.96%) with a loss of more than 2 points in the hemoglobin levels. In 18 cases (11.39%), compartment syndrome developed. A total of 14 major amputations (8.86%) and three minor amputations (1.9%) were performed. Major amputations were performed in seven cases of Rutherford class IIA and in seven cases of Rutherford class IIB. During hospitalization (median duration of 6 [4–9.25] days), 13 patients (8.22%) died, one suffering a major amputation; there was one case of Rutherford class Il; and there were four cases of Rutherford class IIA and seven cases of Rutherford class IIB. Upon discharge, the ankle brachial index was measured in 133 cases, with a median value of 0.88 [0.78–0.99].

In the open surgery group, the complications during hospitalization were as follows: 31 local hematomas (10.36%), 23 wound infections (7.69%), and 11 cases of digestive hemorrhage (3.67%). There were 14 (4.68%) major bleeding complications due to intraoperative situations with a decrease of more than 2 points in the hemoglobin levels. In this group, we performed 31 major amputations (10.36%) and five minor amputations (1.67%). The median duration of hospitalization was 8 days (3 and 11.25 days). In-hospital mortality was 12.04% (36 patients). The ankle brachial index measured for this group had a median value of 0.76 [0.68–0.99]

The differences between the two groups in terms of major amputations and in-hospital mortality was without statistical significance.

The 1-month outcomes indicated that 144 patients (91.13%) were alive in the thrombolysis group. Within the first month after the intervention, one patient (with a major amputation) (Rutherford class IIA) died, and three major amputations (two cases of Rutherford class IIA and one case of Rutherford class IIB) were performed. Eight cardiovascular events were recorded—four myocardial infarctions, two atrial fibrillations, and two cases of angina pectoris. At the 1-month follow-up, the ankle brachial index was measured in 129 patients, with a median value of 1 [0.88–1].

In the open surgery group, 263 patients were alive, without any deaths in the first month. We had five major amputations (1.67%), with one not related to the disease, being traumatic. The following cardiovascular events were recorded: acute coronary syndrome—seven patients, three patients with atrial fibrillation, and five patients with myocardial infarction.

At the 3-month follow-up, we evaluated 138 patients in the thrombolysis group. The ankle brachial index was measured in 127 cases, with a median value of 1 [0.97–1]. Six patients died within the 1-month and 3-month follow-ups.

In the open surgery group, we evaluated 259 patients, four patients being dead. Only five acute coronary syndrome complications were noted with medical treatment remission. The median value of the ankle brachial index was 0.86 [0.79–0.98].

One hundred and thirty-four patients were evaluated at 6 months in the thrombolysis group. The ankle brachial index was measured in 123 patients at the 6-month follow-up, with a median value of 1 [0.99–1]. A statistically significant increase in the ankle brachial index was observed in all the assessed patients (F_3_ = 489.69, *p* < 0.0001). Pairwise comparisons revealed a significant increase in the ankle brachial index from the pre-intervention values to each follow-up (*p* < 0.05).

In the open surgery group, we evaluated 253 patients at 6 months. Six patients died in the interval between 3 and 6 months. The median value of the ankle brachial index was 0.87 [0.78–1].

The amputation-free survival rate one month after the thrombolysis was 82.3% (SE 0.03). The univariate analysis of amputation-free survival at 1 month showed that coronary disease (*p* = 0.02), complications after thrombolysis (hemorrhagic complications and compartment syndrome) (*p* = 0.02), and performance of additional procedures in order to obtain distal perfusion (*p* = 0.04) were associated with major amputation and mortality at 1 month. When entering age > 75 years, coronary disease, a history of previous vascular interventions, thrombolysis duration, complications, and performance of additional procedures into the Cox hazard regression analysis, only the complications because of thrombolysis (HR 2.47, 95% CI 1.176–5.219, *p* = 0.01) were predictive of negative outcomes (major amputation and mortality). The performance of additional procedures to obtain distal perfusion was predictive of an improved outcome at 30 days (HR 0.34, 95% CI 0.13–0.904, *p* = 0.03).

**Figure 1:**
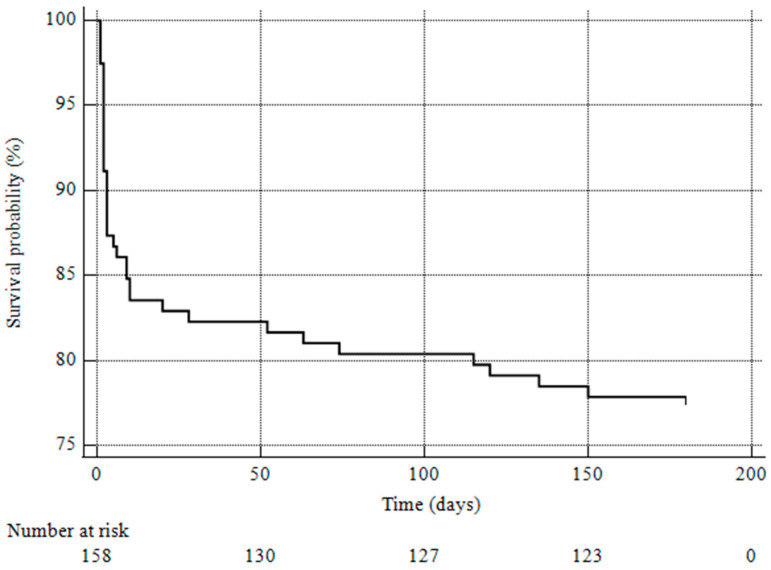
Kaplan–Meier curve showing amputation-free survival rate.

At 6 months, the amputation-free survival rate was 77.85% (SE 0.033) (Figure 1). The univariate analysis of amputation-free survival at 1 month showed that the presence of coronary disease (*p* = 0.02) was associated with major amputation and mortality at 1 month. When entering coronary disease, complications, and performance of additional procedures into the Cox hazard regression analysis, only coronary disease (HR 2.78, 95% CI 1.249–6.200, *p* = 0.01) was predictive of negative outcomes (major amputation and mortality) at 6 months.

**Figure 2:**
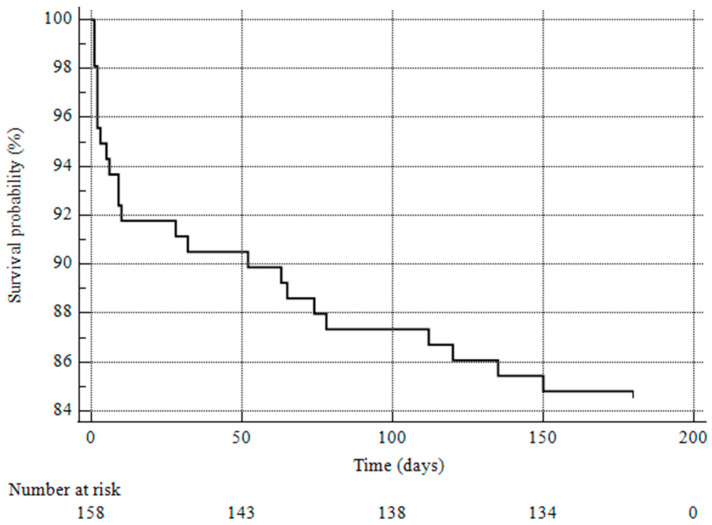
Kaplan–Meier curve showing 6-month overall survival rate.

The estimated survival rate at 6 months was 84.81% (SE 0.02) (Figure 2). The mean survival time was 158.74 days (95% confidence interval 150.41–167.09 days). The univariate analysis showed that coronary disease (*p* = 0.03) and COPD (*p* = 0.01) were associated with an increased risk of death at 6 months. When entering these variables into the Cox hazard regression analysis, only coronary disease (HR 3.3, 95% CI 1.302–8.379, *p* = 0.01) was found to significantly increase the risk of death at 6 months.

In the open surgery group, the amputation-free survival rate at 1 month was 89.63%, and at 3 months it was 78.35%, without statistically significant differences between the groups. Also, in the open surgery group, when entering different variables, we observed that the presence of coronary disease and the duration of ischemia were associated with an increased risk of death both at 30 days and at 3 and 6 months (*p* = 0.02 for coronary disease and 0.01 for ischemia duration).

## 4. Discussion

The 2020 European Society for Vascular Surgery guidelines for acute limb ischemia recommend CDT as the first-line treatment for Rutherford IIa patients [5]. The guidelines also take into consideration this procedure in patients with Rutherford IIb at the recommendations level [7,12], but there are not sufficient studies to prove this. An important aspect to mention is patients with stage Ia. Although the guidelines do not recommend intervention in patients with sudden onset claudication because they respond well to conservative treatment, it must be considered when these patients have an active life and claudication affects their quality of life [13]. In a systematic review published in the EJVES, the authors reiterated that in non-disabling claudication, conservative treatment is recommended in stage I patients. Furthermore, they considered not applying thrombolysis procedures but only certain procedures such as percutaneous thrombectomy or thromboaspiration, in particular because of the increased risk of bleeding. However, the study could not compare high-dose versus low-dose regimens, mentioning that high-dose regimens are associated with higher risks [14].

Furthermore, the systematic review argued that further studies are needed to get a clear indication for treatment of these patients [14]. Furthermore, as mentioned, the stage I patients we revascularized had active lifestyles, and claudication affected their quality of life. This phenomenon needs to be investigated in randomized trials, especially in the context of the many endovascular treatment options currently available.

In our study, the outcomes were limb salvage and mortality at 6 months.

Regarding limb salvage, the results of our study showed a success rate of 82.3% at 30 days and 77.85% at 6 months. The limb salvage rates were influenced by hemorrhagic complications and compartment syndrome because of reperfusion.

In a systematic review of the literature [7], 106 studies were identified with different thrombolytic regimens, of which 17 were on tPA regimens. The mean treatment duration ranged between 17.3 and 32.7 h. For the rt-PA regimens, the major bleeding complications rate was 18.6% [7], which compares with a rate of 6.96% in our study, leading to the conclusion that the use of our protocol is safe.

In the literature, there is a debate about adding heparin to the thrombolytic regimen. Some authors recommend continuous systemic heparinization with a target-activated partial thromboplastin time of 1.5–2 times the baseline. In some studies, this regimen was associated with an increase in major bleeding [15]. Berridge et al. investigated the effect of intra-arterial administration of heparin in a regimen of 250 IU/h, but they did not notice any advantage [16].

We think that heparin administration on the catheter sheath can improve outcomes by preventing sheath thrombosis.

Our rate of hemorrhagic complications comprised only five cases of major complications (gastrointestinal and cerebral ones). These kind of major bleeding complications required cessation of treatment and increased the amputation rate.

In our study, we had 28 cases of local hematomas, only eight of them requiring an additional surgery procedure for evacuation of the hematoma and arterial repair. Local hematomas may be driven by the administration of heparin through the sheath, but none of the local complications negatively influenced the outcomes of the patients; therefore, the utility of sheath heparinization should be further investigated.

Another important issue is that 81.01% of the patients presented more than 24 h after symptom onset. We tried thrombolysis after more than 14 days, all patients having preserved motility and sensibility. Only 15.78% of the patients with more than 14 days after symptom onset suffered a major amputation. It is possible that these patients had well-developed collateral circulation, thus making the leg structures more resistant to the ischemic event [17,18].

The performance of additional procedures to obtain distal perfusion was predictive of an improved outcome at 6 months (HR 0.38, 95% CI 0.164–0.879, *p* = 0.02), and was found to be predictive of a decreased risk of death (HR 0.13, 95% CI 0.031–0.570, *p* = 0.006).

When speaking about the late survival rate, this was close to 84% at 6 months and was influenced only by the presence of coronary disease and not by factors related to the thrombolysis episode. In the systematic review conducted by Ebben et al. [7], late mortality rates were reported in 30% of the studies included and were around 15%. Only 53% of the studies reported the cause of death, and we consider it important to identify if there is a correlation between the determinants of thrombolysis and a late potentially fatal outcome, especially since we are discussing a population that has multiple comorbidities and an altered life expectancy because of cardiac risk factors [19]. Within the range of 1 month after thrombolysis, the factors influencing mortality were caused by complications such as hemorrhagic events and compartment syndrome.

When discussing the comparison between open and endovascular treatment in terms of major amputations, in a retrospective study comparing the two techniques for stage II ALI, the rate of major amputations was 10% for open vs. 7.2% for endovascular techniques at 30 days after the procedure [9].

In another study of 322 patients, the primary patency rates were 87.1% for hybrid procedures vs. 66.3% for open embolectomy, which supports the primary endovascular approach in these patients, especially in the case of pre-existing chronic arterial lesions [20].

Another aspect that should be mentioned is the type of thrombolytic agent used. There are studies mentioning urokinase as an effective agent for thrombolysis in low-dose regimens. In our hospital, we have only rt-PA available and not urokinase, but we consider that thorough studies are needed to compare the different types of regimens to choose what is optimal for the patients [21].

When discussing comparisons with the data in the literature regarding open versus endovascular treatment, a 2020 study conducted by Kolte et al. on 10,484 hospitalizations for acute ischemia concluded that patients treated endovascularly had lower in-hospital mortality but more vascular complications compared to those treated surgically. They also found, similar to what we identified, that amputation rates between the two groups were similar, but their study only compared in-hospital data without follow-up [22].

Another study carried out in 2019 comparing the two types of treatment concluded that both treatment methods are safe for patients according to the limb salvage rates and acceptable survival rates but that there is no clear preference for one of the techniques [23].

A cohort analysis in Sweden of 16,229 procedures broadly identified that both techniques are safe for patients, with approximately equal limb salvage curves [12].

This study’s limitations are the small sample group and the retrospective data collection. In addition, the follow-up was also limited because of the rise of the COVID-19 pandemic [24]. Another limitation, this time regarding the procedures, is linked to the fact that additional procedures were limited to balloon PTA and stent PTA (without other endovascular procedures like mechanical thrombectomy or atherectomy).

## 5. Conclusions

In the light of our results, we can advocate the usage of a thrombolytic regimen in patients with a life expectancy of more than 6 months, even in Rutherford stage IIb patients, if there is no major impairment in the sensorial and mobility function of the ischemic leg.

There is a large variety of regimens for thrombolysis, and we are in need of a standardized regimen and prospective studies in order to assess the best regimen to use.

We also know that ALI is associated with a high complication rate even after successful revascularization, and we need to find the best therapeutic strategies and interdisciplinary consensus to address them.

Moreover, regarding the differences between the two groups, we can say that there were no significant differences in the results in the patients treated with the two techniques. However, how do we choose the best option for the patient? At this moment, we consider that randomized trials are needed to have this answer.

Standardization of practice should be an important goal, and performing critical tasks in the same manner all the time can reduce the mistakes any doctor might make.

## Figures and Tables

**Table 1:** Baseline characteristics of the patients.

Variable	Thrombolysis Group	Open Surgery Group
Age, years (mean ± SD)	64.99 ± 11.91	72.87 ± 12.43
Sex		
Males, n (%)	114 (72.15)	191 (63.88%)
Females, n (%)	44 (27.85)	108 (36.12%)
Risk factors		
Arterial Hypertension, n (%)	132 (83.54)	274 (91.63%)
Hyperlipidemia, n (%)	104 (65.82)	193 (64.55%)
Diabetes mellitus, n (%)	49 (31.01)	86 (28.76%)
Chronic kidney disease, n (%)	19 (12.02)	45 (15.05%)
Atrial fibrillation, n (%)	55 (34.81)	202 (67.60%)
COPD, n (%)	14 (8.86)	41 (13.72%)
Cardiac insufficiency, n (%)	63 (39.87)	201 (67.22%)
Coronary disease, n (%)	20 (12.65)	205 (68.56%)
Smokers, n (%)	81 (51.27)	178 (59.53%)
Previous vascular interventions		
Embolectomy, n (%)	24 (15.19)	31 (10.36%)
Bypass, n (%)	30 (24.68)	42 (14.04%)
Balloon angioplasty, n (%)	5 (3.16)	8 (2.67%)
Stent angioplasty, n (%)	8 (5.06)	3 (1%)
Pre-admission treatment		
Anticoagulant, n (%)	22 (13.92)	174 (58.19%)
Antiplatelet, n (%)	54 (34.17)	204 (68.22%)
Anticoagulant and antiplatelet, n (%)	40 (25.32)	56 (18.72%)
Rutherford classification of ischemia severity		
I, n (%)	18 (11.39)	8 (2.67)
IIA, n (%)	77 (48.74)	176 (58.86%)
IIB, n (%)	63 (39.87)	115 (38.46%)

## Data Availability

The original contributions presented in the study are included in the article/Supplementary Materials, further inquiries can be directed to the corresponding author.

## Supplementary Materials

The following supporting information can be downloaded at: https://www.mdpi.com/article/10.3390/life14111516/s1, Table S1: Thrombolysis Database.
